# Quality of life and functional outcome after infravesical desobstruction and HIFU treatment for localized prostate cancer

**DOI:** 10.1186/s12894-017-0198-2

**Published:** 2017-01-11

**Authors:** G. Hatiboglu, I. V. Popeneciu, M. Deppert, J. Nyarangi-Dix, B. Hadaschik, M. Hohenfellner, D. Teber, S. Pahernik

**Affiliations:** Department of Urology, University of Heidelberg, Im Neuenheimer Feld 110, 69120 Heidelberg, Germany

**Keywords:** HIFU, Quality of life, Outcome

## Abstract

**Background:**

To evaluate quality of life, functional and oncological outcome after infravesical desobstruction and HIFU treatment for localized prostate cancer.

**Methods:**

One hundred thirty-one patients, treated with TURP and HIFU in a single institution were followed up for oncological and functional outcome. Oncological outcome was quantified by biochemical recurrence free survival using the Stuttgart and Phoenix criteria. Quality of life was assessed by usage of standardized QLQ-C30 and QLQ-PR25 questionnaires. In addition, functional questionnaires such as IPSS and IIEF-5 were used. Complications were assessed by the Clavien-Dindo classification.

**Results:**

One hundred thirty-one patients with a mean age of 72.8 years (SD: 6.0) underwent HIFU for prostate cancer (29.0% low risk, 58.8% intermediate risk, 12.2% high risk). PSA nadir was 0.6 ng/ml (SD: 1.2) after a mean of 4.6 months (SD: 5.7). Biochemical recurrence free survival defined by Stuttgart criteria was 73.7%, 84.4% and 62.5% for low-, intermediate- and high-risk patients after 22.2 months. Complications were grouped according to Clavien-Dindo and occurred in 10.7% (grade II) and 11.5% (grade IIIa) of cases. 35.1% of patients needed further treatment for bladder neck stricture. Regarding incontinence, 14.3%, 2.9% and 0% of patients had de novo urinary incontinence grade I°, II° and III° and 3.8% urge incontinence due to HIFU treatment. Patients were asked for the ability to have intercourse: 15.8%, 58.6% and 66.7% of patients after non-, onesided and bothsided nervesparing procedure were able to obtain sufficient erection for intercourse, respectively. Regarding quality of life, mean global health score according to QLQ-C30 was 69.4%.

**Conclusion:**

HIFU treatment for localized prostate cancer shows acceptable oncological safety. Quality of life after HIFU is better than in the general population and ranges within those of standard treatment options compared to literature. HIFU seems a safe valuable treatment alternative for patients not suitable for standard treatment.

## Background

High intensified focused ultrasound (HIFU) is a minimal invasive, thermoablative treatment option for patients with localized prostate cancer. Its aim is equivalent oncological safety with reduced toxicity, compared to standard treatment options [1]. HIFU can be performed in general or spinal anesthesia via transrectal approach. Focused, high energetic ultrasound waves cause thermal alteration and cavitation, causing coagulative necrosis and thereby destroying malignant tissue [2, 3].

Since the initial presentation in 1995 [4], several studies have evaluated oncological and functional outcome after HIFU. Recent publications report of 76%, 63% and 57% biochemical free survival after 8 years for low-, intermediate- and high-risk patients [5]. The 10-year prostate cancer specific survival rate and metastasis-free survival rate were 97% and 94%, respectively [5]. Regarding morbidity of HIFU treatment, severe incontinence rates of 3.1% and erectile function preservation of up to 42.3% are described [5]. Patients rejecting standard treatment and preferring HIFU do this with the expectation for less complications and less invasiveness compared to standard treatment. Especially incontinence, erectile dysfunction after radical prostatectomy as well as gastrointestinal and genitourinary side effects after radiotherapy are feared by many patients and can impair their quality of life [6]. Recent studies have evaluated quality of life for prostate cancer patients after local therapy, showing good results with moderate alteration in erectile and lower urinary tract function with minimal decrease in quality of life [7, 8]. The authors utilized standardized questionnaires for this evaluation like the European Organisation for Research and Treatment of cancer (EORTC) quality of life questionnaire QLQ-C30 and the prostate specific module QLQ-PR25. The QLQ-C30 questionnaire evaluates overall health related quality of life as well as several functional domains and general cancer related symptoms. The QLQ-PR25 assesses urinary symptoms, sexual activity and function, as well as bowel symptoms. Both questionnaires have been evaluated and validated extensively [9, 10]. Regarding quality of life for prostate cancer patients, both questionnaires are routinely used.

To our knowledge, data about quality of life after HIFU therapy using standardized questionnaires are rare and have not been evaluated in a standardized fashion so far. The aim of the study was to investigate prospectively quality of life after HIFU ablation of the prostate for the local treatment of prostate cancer.

## Methods

One hundred thirty-one patients undergoing infravesical desobstruction and HIFU treatment for localized prostate cancer between 02/2008 and 12/2012 were followed up in a prospectively conducted database. The study protocol was approved by the ethics committee of the University of Heidelberg (S-182/2012). All patients gave written informed consent.

All patients were treated with the Ablatherm HIFU device (Ablatherm integrated imaging device; EDAP-TMS, Vaulx-en-Velin, France). Before HIFU treatment an infravesical desobstruction was routinely done one day prior to HIFU treatment, normally by transurethral resection of the prostate (TURP) or by greenlight laservaporisation of the prostate [11]. The mode of infravesical desobstruction was at patients’ preference (TUR-P was recommended, some patients explicitly wished to undergo greenlight laservaporisation). HIFU treatment was performed as inpatient procedure in combined spinal and epidural anesthesia. Nervesparing procedure was only done in patients explicitly demanding for this. All these patients were intensively informed about higher risk for recurrent prostate cancer but wished to undergo nervesparing anyway and with awareness of all risks (this was also documented in informed consent). In these cases, mainly one sided nervesparing was done - if prostate biopsy showed cancer infiltration on only one side the nervesparing was done on the contralateral side. However, some patients explicitly wished both sided nervesparing, taking into account the risk for tumor recurrence. For all patients, the following parameters were assessed and entered in a prospective conducted database: patient age, body mass index, prostate volume, PSA value at diagnosis, Gleason Score, clinical stage, risk classification (according to D’Amico et al. [12, 13]), PSA nadir and time to PSA nadir, biochemical recurrence according to Stuttgart criteria [14] and Phoenix criteria [15]. In addition, treatment related data were evaluated and included: type of preoperative infravesical desobstruction, duration and number of applied lesions during HIFU treatment, nervesparing (non-,unilateral-,bilateral nervesparing), hospital stay, indwelling time for transurethral and suprapubic catheter, uroflowmetry and residual volume (pre-HIFU and 2 weeks post-HIFU). In addition, complication were evaluated according to Clavien-Dindo [16]. Patients were asked to fill out the international prostate symptom score (IPSS) and international index for erectile function (IIEF-5) questionnaire before and 3 months after HIFU treatment. Continence was evaluated and graduated during follow-up, the usage and number of used pads was evaluated. Patients were also asked for sexual activity after HIFU treatment including the usage of adjuvant medication for erection.

### Assessment of quality of life

All patients were asked to complete the European Organisation for Research and Treatment of cancer (EORTC) quality of life questionnaire QLQ-C30 (Version 3.0) and the prostate specific module QLQ-PR25 retrospectively in 12/2012. Both questionnaires are internationally validated and used in cancer patients. They were used in German translation. The QLQ-C30 questionnaire consists of 30 questions, the QLQ-PR25 of 25 questions. The QLQ-C30 measures global health related quality of life, five functional domains (physical, role, emotional, cognitive and role function) and nine symptom scales (fatigue, nausea and vomiting, pain, dyspnoe, insomnia, appetite loss, constipation, diarrhea, financial difficulties). The QLQ-PR25 questionnaire, consisting of 25 questions, assesses urinary symptoms, bowel symptoms, treatment-related symptoms and sexual symptoms and functioning. In both instruments, all answers ranged from 1 (not at all) to 4 (very much), despite 2 questions in the QLQ-C30 considering global health related quality of life ranging from 1 (very poor) to 7 (excellent). Both questionnaires have been interpreted according to the EORTC guidelines. The scores have been converted to linear scales ranging from 0 to 100. Higher values in functional scales and health related quality of life represent better results. Lower values in symptom scales represent less symptoms.

### Statistical analysis

Above mentioned variables were evaluated by descriptive statistics. Median, mean, standard deviation and range were calculated for every variable using Microsoft Excel and IBM SPSS statistics version 20.

## Results

One hundred thirty-one patients with a mean age of 72.8 ± 6.0 years have been included to this analysis. PSA values at prostate cancer diagnosis were 9.6 ± 14.9 ng/ml. Gleason score was 6 in 57 patients (43.5%), 7 in 66 patients (50.4%) and ≥ 8 in 8 patients (6.1%). 29.0% of patients had a low risk, 58.8% an intermediate risk and 12.2% a high risk prostate cancer (Table 1). When presenting for HIFU treatment, 28 patients (21.4%) have been on neoadjuvant hormonal therapy, that has been stopped directly after HIFU treatment. Bothsided nervesparing was performed in 21.1%, 9.6% and 6.7%, onesided nervesparing in 28.9%, 26.0% and 6.7% of low-, intermediate- and high-risk patients, respectively. After HIFU the mean PSA nadir was 0.6 ± 1.2 ng/ml (median: 0.1 ng/ml; range 0.01-6.50 ng/ml) and reached after 4.7 months (SD: 5.7) (Table 2). Mean follow up was 22.2 months (SD: 16.1). 28 patients (21.4%) developed a biochemical recurrence, defined as PSA Nadir + 1.2 ng/ml (according to Stuttgart criteria [14]) after a mean time of 15.5 ± 11.6 months. Among them, 10 patients (26.3%), 12 patients (15.6%) and 6 patients (37.5%) of low-, intermediate-and high-risk profile, respectively. Therefore, biochemical recurrence free survival was 73.7%, 84.4% and 62.5% for low-, intermediate- and high-risk patients at latest follow-up. In addition, 3 patients had histological confirmed recurrence that was confirmed in prostate biopsy in patients undergoing biopsy after 6 months at own wish or after bladder neck resection due to infravesical obstruction.

**Table 1:** Demographics

	n (%)	mean (SD)
Age (years)	131	72.8 (6.0)
Clinical stage		
cT1	83 (63.8%)	
≥ cT2	47 (36.2%)	
Gleason Score		
≤ 6	57 (43.5%)	
7	66 (50.4%)	
≥ 8	8 (6.1%)	
inital PSA (ng/ml)	130	9.6 (14.9)
Risk stratification (D’Amico)		
Low - risk	38 (29.0%)	
Intermediate - risk	77 (58.8%)	
High - risk	16 (12.2%)	
Prostate volume (ml)	130	43.7 (25.6)
Prostate volume after resection	123	26.0 (12.5)

**Table 2:** Perioperative parameter

	n (%)	mean (SD)
Treated HIFU volume (ml)	126	27.7 (9.9)
Duration of HIFU treatment (minutes)	127	104.3 (28.8)
Applied lesions	127	433.8 (136.2)
Nervesparing		
No nervesparing	79 (60.3%)	
One-sided nervesparing	31 (23.7%)	
Both-sided nervesparing	16 (12.2%)	
Data missing	5 (3.8%)	
Transurethral catheter indwelling time	125	4.0 (2.5)
Suprapubic catheter indwelling time	78	14.5 (15.0)

Of these 31 patients with recurrent prostate cancer, 18 patients (58.1%) underwent active therapy. Twelve of these patients were lost to follow-up. Five patients underwent salvage radiotherapy, two 2 patients salvage-HIFU therapy, one patient prostatectomy and one patient brachytherapy and pelvic lymphadenectomy. The other 9 patients (29.0%) did not wish salvage therapy and received antiandrogen treatment. During follow-up, 7 patients (5.3%) deceased – 1 patient because of systemic progression of the prostate cancer, the other 6 patients due to cardiovascular events (Table 3).

**Table 3:** Oncological outcome

	n (%)	Mean (SD)
PSA – nadir (ng/ml)	101	0.6 (1.2) median: 0.1 (range: 0.01–6.50)
Time to nadir (months)	99	4.7 (5.7)
Recurrencies	31 (23.7%)	
Biochemical recurrence (BCR) Suttgart criteria	28 (21.4%)	
low - risk - PCa	10 (7.6%)	
intermediate - risk – PCa	12 (9.2%)	
high - risk – PCa	6 (4.6%)	
Time to BCR (in months) (Stuttgart criteria)	28	15.5 (11.6)
Time to BCR (in months) (Phoenix criteria)		17.4 (12.5)
low - risk - PCa	10	14.1 (8.2)
intermediate - risk - PCa	12	19.8 (13.8)
high - risk – Pca	6	11.3 (5.4)
Recurrence without BCR	3 (2.3%)	

When defining biochemical recurrence according to Phoenix criteria, 20 patients (15.5%) showed biochemical recurrence with a mean time to recurrence of 17.4 ± 12.5 months. Biochemical recurrence free survival was therefore 81.6%, 89.6% and 68.8% for low-, intermediate- and high-risk patients at latest follow-up, respectively.

Regarding treatment data, 109 patients (83.2%) underwent TUR-P, 18 patients greenlight-laser vaporization (13.7%) and 1 patient (0.8%) combination of both in advance to HIFU treatment. Resection was done the day prior to HIFU. Mean prostate size was thereby reduced from 43.7 ml (±25.6) to 26.0 ml (±12.5). Three patients (2.3%) had no prior infravesical desobstruction. During HIFU treatment, a one-sided nervesparing was done in 31 patients (23.7%), a both sided nervesparing in 16 patients (12.2%). Perioperative side effects up to 30 days after HIFU treatment were assessed and grouped according to Clavien-Dindo classification and occurred in 25 patients (19.1%), 4 patients had 2 complications. Main complications were infravesical obstruction and urinary tract infections. With respect to infravesical obstruction, grade II and grade IIIa complications occurred in 14 (10.7%) and 15 (11.5%) cases, respectively. Regarding late complications beyond 30 days, 47 patients (35.9%) needed further treatment. 46 patients (35.1%) needed catheterization or suprapubic catheter insertion and transurethral resection because of bladder neck obstruction. One patients needed DJ insertion because progression of prostate cancer.

IPSS score before and after HIFU treatment was 10.9 ± 7.1 and 9.2 ± 6.2, respectively. Uroflowmetrie before and after HIFU treatment showed max. flow of 14.0 ml/s (SD 8.2) and 14.1 ml/s (SD 8.7) with post voiding residual volume of 45.5 ml (SD 69.4) and 54.0 ml (SD 93.6), respectively. Regarding incontinence, 14.3%, 2.9% and 0% of patients had de novo urinary incontinence grade I°, II° and III° and 3.8% urge incontinence due to HIFU treatment. Before HIFU treatment 97.1% of patients did not need a pad. After HIFU treatment, 9.5%, 6.7% and 1.9% of patients needed 1pad, 2pads or >2pads, respectively, meaning 81.9% did not need a pad at all at latest follow-up.

IIEF-5 scores showed 17.2 ± 7.3 and 9.7 ± 8.0 before and after HIFU treatment in all patients, respectively. When grouped according to nervesparing procedure, mean IIEF-5 score before and after HIFU was 15.1 ± 8.1 vs. 7.5 ± 7.4 for the non-nervesparing group, 19.7 ± 4.8 vs. 12.5 ± 8.3 for the one-sided nervesparing group and 20.7 ± 5.3 and 12.1 ± 7.5 for the both sided nervesparing group, respectively. Regarding the ability to have sexual intercourse, 15.8%, 58.6% and 66.7% of patients after non-, onesided and bothsided nervesparing procedure were able to obtain sufficient erection for intercourse, respectively. 33.3% of these patients used PDE5 inhibitors, 3 patients mechanical devices to obtain erection. Functional outcomes after HIFU therapy are displayed in Table 4.

**Table 4:** Functional outcome after HIFU treatment (mean(SD))

	Pre - HIFU	Post - HIFU
Continence and micturation symptoms		
IPSS – Score	10.9 (7.1)	9.2 (6.2)
Micturation volume (ml)	207.7 (116.5)	189.2 (92.4)
Post voiding Residual volume(ml)	45.5 (69.4)	54.0 (93.6)
Qmax (ml/sec)	14.0 (8.2)	14.1 (8.7)
Continence		
Continent	93 (88.6%)	71 (67.6%)
Incontince grade I°	5 (4.8%)	20 (19.0%)
Incontince grade II°	0 (0.0%)	3 (2.9%)
Incontince grade III°	1 (1.0%)	1 (1.0%)
Urge incontinence	6 (5.7%)	10 (9.5%)
Pad usage		
No pads	102 (97.1%)	86 (81.9%)
1 pad	1 (1.0%)	10 (9.5%)
2 pads	1 (1.0%)	7 (6.7%)
> 2 pads	1 (1.0%)	2 (1.9%)
Sexual function		
Sexual intercourse after HIFU		
Yes		36 (35.6%)
No		65 (64.4%)
Due to HIFU treatment		46 (45.5%)
Other reason		55 (54.5%)
IIEF – 5		
*IIEF - 5 – score*	17.2 (7.3)	9.7 (8.0)
IIEF - 5 - *non – nervesparing*	15.1 (8.1)	7.5 (7.4)
IIEF - 5 - *onesided - nervesparing*	19.7 (4.8)	12.5 (8.3)
IIEF - 5 - *bothsided – nervesparing*	20.7 (5.3)	12.1 (7.5)

Quality of life was evaluated using the QLQ-C30 questionnaire and showed a global health status of 69.4% (SD: 20.6). Functional scales revealed physical functioning of 86.2% (SD 17.7) after HIFU treatment. Regarding symptom scales, fatigue and insomnia were the most frequent symptoms with 25.9% (SD: 24.8) and 31.4% (SD: 31.4) of cases in a cohort of men aged 72.8 ± 6.0 years. For further evaluation of prostate specific symptoms the QLQ-PR25 questionnaire was used and showed sexual activity and functioning of 56.9% (SD: 28.1) and 49.6% (SD: 20.5), respectively. Evaluation of symptom scales showed urinary symptoms in 28.3% (SD: 18.4) and urinary bother in 38.6% (SD: 35.6) of patients after HIFU. Bowel symptoms did appear in 6.7% (SD: 11.2) of patients. The overall quality of life did not change before and after HIFU (evaluated by the IPSS questionnaire). All results are displayed in Table 5.

**Table 5:** Quality of life – post-HIFU outcome of QLQ-C30, QLQ-PR25 and IPSS-QoL questionnaires (*n* = 105). Data are shown as mean with standard deviation

QLQ - C30	
Global health status/QoL (QL2)	69.4 (20.6)
Functional scales	
Physical functioning (PF2)	86.2 (17.7)
Role functioning (RF2)	79.0 (29.9)
Emotional functioning (EF)	74.8 (23.5)
Cognitive functioning (CF)	83.3 (20.5)
Social functioning (SF)	74.8 (25.0)
Symptom scales/items	
Fatigue (FA)	25.9 (24.8)
Nausea and vomiting (NV)	1.5 (6.7)
Pain (PA)	17.1 (27.8)
Dyspnoea (DY)	18.1 (26.6)
Insomnia (SL)	31.4 (31.4)
Appetite loss (AP)	4.8 (16.9)
Constipation (CO)	16.2 (27.0)
Diarrhoea (DI)	7.3 (19.6)
Financial difficulties (FI)	10.5 (23.3)
QLQ - PR25	
Functional scales	
Sexual activity(PRSAC)(*n* = 104)	56.9 (28.1)
Sexual functioning (PRSFU)(*n* = 71)	49.6 (20.5)
Symptom scales/items	
Urinary symptoms (PRURI)	28.3 (18.4)
Urinary bother (PRAID)	38.6 (35.6)
Bowel symptoms (PRBOW)	6.7 (11.2)
ADT – treatments symptoms (PRHTR)	16.4 (16.4)
Quality - of - life; IPSS	
Prä - HIFU	2.1 (1.4)
Post - HIFU	2.0 (1.3)

## Discussion

The present study was performed to evaluate quality of life after HIFU treatment for prostate cancer. To our knowledge, this is the first study investigating this topic. Quality of life was assessed by standardized questionnaires. In addition, functional outcome and oncological results were evaluated with respect to limited followup.

Regarding oncological results, recent reports from Crouzet et al. showed 7 years biochemical free survival (Phoenix Criteria) in 75%, 63% and 62% for low-, intermediate- and high-risk prostate cancer patients [5]. Using the recently supposed stricter Stuttgart criteria (meaning PSA nadir +1.2 ng/ml [14]), Pfeiffer et al. described 5 years biochemical free survival as 85%, 65% and 55% for low-, intermediate- and high-risk prostate cancer [17]. The present series mainly consisted of intermediate risk patients (58.8%) because high risk patients were consulted for standard treatment and low risk patients for active surveillance. In this cohort, biochemical recurrence free survival was 76.3%, 84.4% and 64.7% (using Stuttgart criteria) and 81.6%, 89.6% and 68.8% (using Phoenix criteria) for low-, intermediate- and high-risk patients with a mean follow up of 22.2 months. However, 3 patients of our cohort also had histological proven recurrence without biochemical recurrence. Previous studies by Ganzer et al. showed, that negative biopsy rates correlate with postoperative PSA nadir. Ganzer et al. described negative biopsy rates of 91.6% for patients with PSA nadir ≤ 0.2 ng/ml. Thus, 8.4% of patients had remaining prostate cancer tissue in control biopsies after 3–6 months [14] with a PSA value < 0.2 ng/ml.

In our cohort, side effects were limited in severity. Main complications were urinary tract infections and infravesical obstruction. While infections were mainly managed successfully by antibiotic treatment, 25% of patients needed further surgical treatment in form of transurethral resection of bladder neck strictures. These complications and rates were also described in other publications as the most frequent re-interventions [18–20]. More severe complications like fistulas did not occur in our cohort. Regarding postoperative micturition and continence, IPSS and uroflowmetry data did not change after HIFU treatment. Previous studies could show, that combining TURP and HIFU is mandatory to reduce postoperative voiding dysfunction [21]. However, even previous TURP cannot reduce infravesical obstruction completely. During follow-up, 35.1% of patients needed further treatment for infravesical obstruction after HIFU treatment. Previous studies confirm, that bladder neck obstruction or urethral strictures are the most common side effects of HIFU treatment [5, 18]. Crouzet et al. described up to 16% bladder neck obstruction in their group, when TUR-P and HIFU were combined in one session. To reduce these side effects, an interval of up to 6 weeks between TUR-P and HIFU has been proposed [11] with reduction of infravesical obstruction to 11%, as described by Crouzet et al. [5]. Compared to our cohort, median pre-HIFU prostate volume was smaller in the cohort described by Crouzet et al. (22–24.5 ml) compared to our cohort (38.5 ml), which could explain the higher bladder neck obstruction rates in the present study due to a selection bias.

Evaluation of erectile function in our group revealed, that postoperative erectile function depends on nervesparing procedure. Most studies do not report of nervesparing as this means sparing a few millimeters of prostatic tissue and therefore increasing the risk for biochemical recurrence [22]. As expected, the PSA nadir ranges were higher (up to 6.5 ng/ml), most likely because of nervesparing procedure, that has been done in 35.9% of patients. Warmuth et al. [18] reviewed 20 studies with a total of 3018 patients undergoing whole gland ablation and reported of erectile dysfunction rates ranging from 20–39%. Crouzet et al. [5] recently reported their results of 1002 patients and described preservation of erectile function in 42.3% patients (<70 years: 55.6%; >70 years: 25.6%). None of these trials took nervesparing procedure into account. In our cohort, erectile function could be preserved in only 15% in patients undergoing whole gland ablation. These patients tended to be of advanced age (mean age in non-nervesparing group was 74.4 years), that is known to be associated with impaired baseline potency status [23] and therefore these patients normally do not wish nervesparing procedure. In contrast, both sided nervesparing HIFU could preserve erectile function in up to 66% of our patients (median age in this group was 69.7 years).

Patients undergoing HIFU ablation instead of standard treatment commonly expect less side effects and better preservation of quality of life. As quality of life represents a multidimensional construct that includes physical, social, psychological and functional domains, its assessment needs complex and standardized questionnaires. Shoji et al. examined the changes in quality of life for patients at a mean age 68 years undergoing HIFU by using the japanese versions of the FACT-G and FACT-P questionnaires (functional assessment of cancer therapy – general and prostate cancer module) [24]. The authors reported, that general quality of life improves after HIFU therapy while the values for FACT-P questionnaire did not change. However, patients undergoing HIFU may be biased as they actively rejected standard therapy for a new, minimal invasive therapy option. We evaluated quality of life after HIFU therapy using the standardized QLQ-C30 and QLQ-PR25 questionnaire. Mean global health score after HIFU treatment was 69.4% for all patients, 66.4% for patients ≤70 years and 70.9% for patients >70 years. Schwarz et al. evaluated QLQ-C30 data for the general german population [25] and described global health scores of 65.6% and 61.5% for patients ≤70 and >70 years, respectively. When compared to the general german population, patients after HIFU treatment show better quality of life with respect to global health scores. Drummond et al. evaluated quality of life after standard therapy in more than 6000 patients within the PiCTure study (prostate cancer treatment, your experience) [8]. They described global health scores ranging from 73.4% for radical prostatectomy and 69.4% for external beam radiation therapy. With respect to the younger patient collective in the PiCTure study, global health score after HIFU treatment in our collective ranged within the scores after standard treatment options described by Drummond et al. Comparing the results of QLQ-PR25 items revealed more urinary symptoms (28.3% for HIFU vs. 19.8% for standard treatment [8]) and more urinary bother (38.6% for HIFU vs. 15.6% for standard treatment [8]) and less sexual activity (56.9% for HIFU vs. 67.8% for standard treatment [8]).

Limitations of the study include short followup. Results predicting oncological safety need longer followup periods, that should be considered in further studies. Another limitation is the missing, preoperative assessment for quality of life to compare changes for this parameter.

## Conclusion

In conclusion, HIFU treatment for localized prostate cancer shows acceptable oncological safety in limited follow up. However, local recurrence is not always indicated by biochemical recurrence. Compared to literature, the quality of life after HIFU showed better results compared to general population of equivalent age. Patients’ satisfaction ranges within those of standard treatment options. Further studies are needed to compare changes in quality of life before and after HIFU therapy. In addition, nearly 25% of patients needed further treatment for bladder neck obstruction. HIFU seems a safe valuable treatment alternative for patients not suitable for standard treatment.

## Abbreviations

EORTC: European Organisation for Research and Treatment of cancer
HIFU: High intensified focused ultrasound
IIEF-5: International index for erectile function
IPSS: International prostate symptom score
QLQ: Quality of life questionnaire
TURP: Transurethral resection of the prostate
